# Supplementary material for: A Multiplex Quantitative Polymerase Chain Reaction for the Rapid Differential Detection of Subgroups A, B, J, and K Avian Leukosis Viruses
Source: Viruses. 2023 Aug 23;15(9):1789. doi: 10.3390/v15091789 (PMC10535029; doi:10.3390/v15091789)
Supplement: Supplementary file 1 [file viruses-15-01789-s001.zip › viruses-2526440-supplementary.pdf]

Table S1. The general organization of the farms

Region	Farm	Breed	Stock of breeding hens	Average age	Breeding methods
Wuhan	Xin Tianmu	Jianghan chicken	4,000	200-400 d	cages
Yichang	Min Da	Shuanglian chicken	1,0000	180-400 d	cages
Suizhou	Hong Xin	E Qinhuang chicken	3,000	180-400 d	cages
Jinzhou	Zheng Hua	Jianghan chicken	6,000	150-400 d	cages
Daye	Jing Xiu	White feather chicken	8,000	150-400 d	cages

Note: Fans were used for longitudinal ventilation in the chicken house based on temperature and other environmental conditions, while fog line spray humidification was adopted for humidity control.

Table S2. Cq values of ALV-A, ALV-B, ALV-J and ALV-K detected by the multiplex qPCR assay with different probe and primer concentrations.

ALV-A						ALV-B					
**Probe concentration (μL)**	**Primer concentration (μL)**					**Probe concentration (μL)**	**Primer concentration (μL)**				
	**0.2**	**0.3**	**0.4**	**0.5**	**0.6**		**0.2**	0.3	0.4	0.5	0.6
0.2	22.427	21.190	22.881	23.728	22.431	0.2	22.322	20.621	22.060	22.820	23.139
0.4	22.032	22.360	22.368	23.332	21.430	0.4	21.862	21.703	23.010	21.668	22.810
0.6	23.696	24.379	21.553	22.742	22.860	0.6	22.138	22.373	21.826	22.401	23.202
0.8	24.280	21.423	22.023	21.992	24.130	0.8	21.113	22.447	20.484	21.492	21.300
1.0	22.652	22.003	22.951	22.056	22.336	1.0	21.671	20.626	22.872	23.251	22.884
**ALV-J**						**ALV-K**					
**Probe concentration (μL)**	**Primer concentration (μL)**					**Probe concentration (μL)**	**Primer concentration (μL)**				
	0.2	0.3	0.4	0.5	0.6		0.2	0.3	0.4	0.5	0.6
0.2	21.809	20.336	22.496	23.662	23.063	0.2	23.287	20.467	23.415	24.262	24.197
0.4	21.258	23.827	23.000	22.346	23.218	0.4	23.303	24.231	23.985	24.721	23.066
0.6	21.696	22.573	21.252	22.338	22.520	0.6	24.553	21.823	21.511	24.009	22.216
0.8	21.789	22.569	20.504	22.365	22.244	0.8	22.689	24.107	23.183	22.690	25.155
1.0	23.743	21.121	23.254	22.920	21.771	1.0	20.989	24.876	24.042	24.161	24.426

Table S3. Cq values of ALV-A, ALV-B, ALV-J and ALV-K detected by the multiplex qPCR assay with different annealing temperatures.

Subtype	Temperature(℃)										
	55	56	57	58	59	60	61	62	63	64	65
ALV-A	23.350	23.016	22.993	23.049	22.939	22.745	22.963	22.925	22.922	23.287	22.965
ALV-B	25.607	25.101	24.656	24.387	23.176	21.339	24.121	23.644	24.184	23.515	23.154
ALV-J	21.718	21.851	21.724	21.542	21.579	21.360	21.604	21.675	21.644	21.443	21.836
ALV-K	22.164	22.223	22.110	22.186	22.093	21.751	22.042	22.320	22.176	22.095	22.031

Table S4. Agreement of the multiplex qPCR with routine PCR, ELISA and virus isolation in the detection of clinical samples.

Multiplex qPCR	PCR			ELISA			Virus isolation		
ALV	Positive	Negative	Total	Positive	Negative	Total	Positive	Negative	Total
Positive	130	6	136	132	4	136	110	26	136
Negative	0	716	716	29	687	716	0	716	716
Total	130	722	852	161	691	852	110	742	852

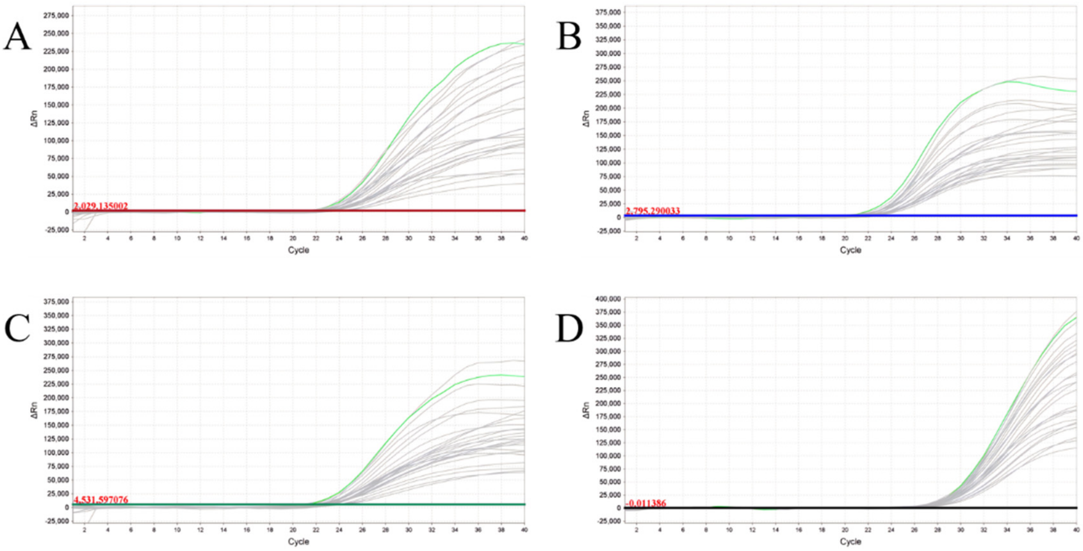

Figure S1. Optimization of primer and probe concentrations of the Multiplex qPCR. A-D: amplification curves (X-axis: Cycle, Y-axis: Fluorescence) of ALV-A, ALV-B, ALV-J and ALV-K detected by multiplex real-time PCR with different probe and primer concentrations. Plasmid standards with concentration of 10^4 copies/μL were chosen as templates for the reactions. The four green lines are the amplification curves of four fluorescence of the most suitable reaction tube.

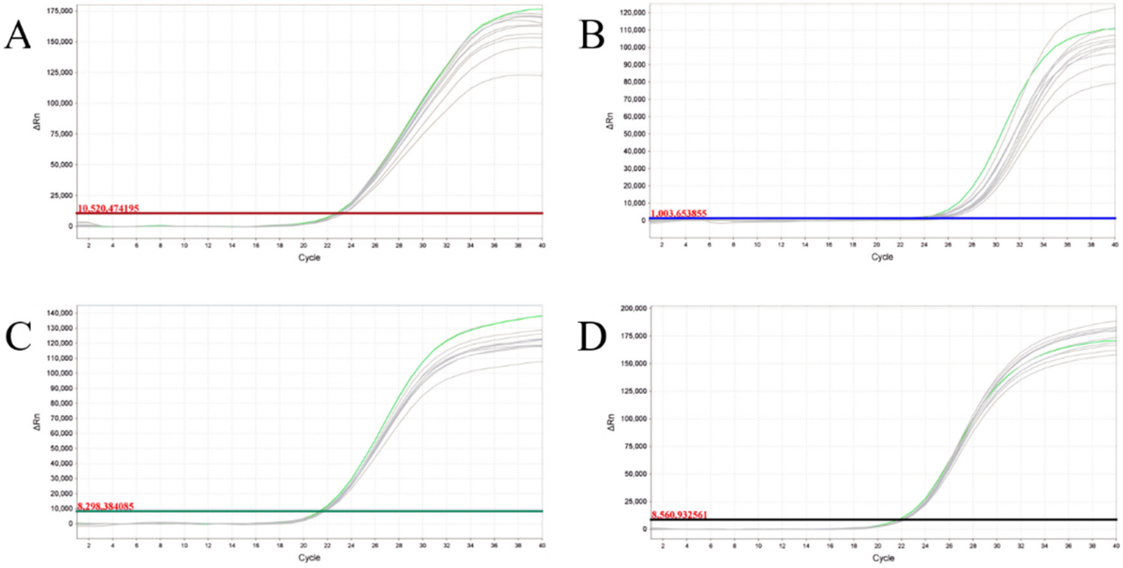

Figure S2. Optimization of annealing temperature of the Multiplex qPCR. A-D: amplification curves (X-axis: Cycle, Y-axis: Fluorescence) of ALV-A, ALV-B, ALV-J and ALV-K detected by multiplex real-time PCR with different temperature. Plasmid standards with concentration of 10^4 copies/μL were chosen as templates for the reactions. The four green lines are the amplification curves of four fluorescence of the most suitable reaction tube.
